# Computer-aided cognitive training combined with tDCS can improve post-stroke cognitive impairment and cerebral vasomotor function: a randomized controlled trial

**DOI:** 10.1186/s12883-024-03613-3

**Published:** 2024-04-19

**Authors:** Yin Chen, Ziqi Zhao, Jiapeng Huang, Tingting Wang, Yun Qu

**Affiliations:** 1 Department of Rehabilitation MedicineInstitute/University/Hospita, West China Hospital of Sichuan University, Chengdu, Sichuan 610041 China; 2 College of Rehabilitation Medicine, West China Hospital of Sichuan University, Chengdu, Sichuan 610041 China; 3 Sichuan Provincial Key Laboratory of Rehabilitation Medicine, Sichuan University, Chengdu, Sichuan 610041 China; 4 Clinical Medical College of Acupuncture Moxibustion and Rehabilitation, Guangzhou University of Chinese Medicine, Guangzhou, Guangdong 510006 China

**Keywords:** Stroke, Post-stroke cognitive impairment, Computer-aided cognitive training, Transcranial direct current stimulation, Cerebrovascular function, Cerebral vasomotor function

## Abstract

**Background:**

Post-stroke cognitive impairment (PSCI) is the focus and difficulty of poststroke rehabilitation intervention with an incidence of up to 61%, which may be related to the deterioration of cerebrovascular function. Computer-aided cognitive training (CACT) can improve cognitive function through scientific training targeting activated brain regions, becoming a popular training method in recent years. Transcranial direct current stimulation (tDCS), a non-invasive brain stimulation technique, can regulate the cerebral vascular nerve function, and has an effect on the rehabilitation of cognitive dysfunction after stroke. This study examined the effectiveness of both CACT and tDCS on cognitive and cerebrovascular function after stroke, and explored whether CACT combined with tDCS was more effective.

**Methods:**

A total of 72 patients with PSCI were randomly divided into the conventional cognitive training (CCT) group (*n* = 18), tDCS group (*n* = 18), CACT group (*n* = 18), and CACT combined with tDCS group (*n* = 18). Patients in each group received corresponding 20-minute treatment 15 times a week for 3 consecutive weeks. Montreal Cognitive Assessment (MoCA) and the Instrumental Activities of Daily Living Scale (IADL) were used to assess patients’ cognitive function and the activities of daily living ability. Transcranial Doppler ultrasound (TCD) was used to assess cerebrovascular function, including cerebral blood flow velocity (CBFV), pulse index (PI), and breath holding index (BHI). These outcome measures were measured before and after treatment.

**Results:**

Compared with those at baseline, both the MoCA and IADL scores significantly increased after treatment (*P* < 0.01) in each group. There was no significantly difference in efficacy among CCT, CACT and tDCS groups. The CACT combined with tDCS group showed greater improvement in MoCA scores compared with the other three groups (*P* < 0.05), especially in the terms of visuospatial and executive. BHI significantly improved only in CACT combined with tDCS group after treatment (*p* ≤ 0.05) but not in the other groups. Besides, no significant difference in CBFV or PI was found before and after the treatments in all groups.

**Conclusion:**

Both CACT and tDCS could be used as an alternative to CCT therapy to improve cognitive function and activities of daily living ability after stroke. CACT combined with tDCS may be more effective improving cognitive function and activities of daily living ability in PSCI patients, especially visuospatial and executive abilities, which may be related to improved cerebral vasomotor function reflected by the BHI.

**Trial registration number:**

The study was registered in the Chinese Registry of Clinical Trials (ChiCTR2100054063). Registration date: 12/08/2021.

## Introduction

Post-stroke cognitive impairment (PSCI) is one of the most common post-stroke dysfunction [1], which greatly reduces the quality of life of patients and increases the disability and mortality rates of patients [2]. The prevalence of PSCI among 10-year stroke survivors is as high as 53.4–61% [3, 4]. Although PSCI is thought to result from damage of cognitive-related neural pathways after stroke, the specific pathogenic mechanisms involved are unknown, leading to a lack of targeted drug therapy [5]. Cognitive training intervention is an effective method to improve PSCI [6]. However, although there have been many studies exploring the effective rehabilitation means for PSCI, there is no consensus on the best treatment for PSCI [7, 8]. Therefore, more effective and diversified treatments need to be explored.

Computer-aided cognitive training (CACT), a method to assist patients in cognitive function training through intelligent training systems and multimedia such as graphics, audio, video and virtual reality technology, began to be applied in the cognitive field as a great substitute or supplement for traditional cognitive rehabilitation [9]. Hsiu-Yu Ho’s study found CACT provided improvement in global cognitive function and specific cognitive domains like working memory, attention and naming [10]. Compared to conventional cognitive training (CCT), CACT is able to provide training at an appropriate level of difficulty based on the patient’s level of cognitive function and provide immediate feedback on the patient’s performance [11], solving problems such as insufficient and uneven distribution of rehabilitation resources and repetitive or boring contents of traditional artificial cognitive training that rely on therapists’ techniques [12]. Transcranial direct current stimulation (tDCS) is a non-invasive neuroregulatory technique, it regulates cortical excitability by applying a weak current to improve PCSI [13]. The left dorsolateral prefrontal cortex (DLPFC) was found to be most closely related to cognitive function [14]. Using anodal tDCS to stimulate DLPFC might show improvement in cognitive impairment.

Previous studies provided the evidence that CACT combined with tDCS could get greater improvement in PSCI than CACT [15–17]. However, research found that CACT in conjunction with tDCS targeting to left DLPFC seemed to show no additional gain compared to the sham stimulation combined with CACT group [18]. Besides, a study suggested that CACT had very limited effects on improving post-stroke cognition function, especially working memory and speed [19]. A meta-analysis showed that tDCS did not seem to improve cognitive domains other than working memory and attention [20], and another study found no evidence of effect of tDCS on cognitive abilities after stroke [21]. Therefore, it is still necessary to study the efficacy of CACT and tDCS and the synergistic effect of CACT combined with tDCS in the treatment of PSCI because their effectiveness is controversial.

In addition, a number of studies have shown that PSCI was closely related to cerebrovascular blood flow velocity, resistance and cerebral vasomotor function [22–24]. Therefore, we also explored the role of CACT and tDCS in hemodynamics and provided evidence of the mechanisms related to cerebrovascular function in the rehabilitation of individuals with cognitive impairment after stroke.

## Materials and methods

This clinical trial is a prospective, single-center, randomized clinical trial that met the CONSORT criteria. This study was approved by the Ethics Committee of West China Hospital of Sichuan University with the number of [2021 (1313)] and registered at the Chinese Clinical Trial Center (ID: ChiCTR2100054063) on December 8, 2021. All included subjects provided written informed consent.

### Participants

Patients with PSCI were recruited from the of the department of rehabilitation medicine of West China Hospital. The inclusion criteria for participants in the study were as follows: (a) diagnosed with ischaemic stroke [25] and confirmed by head CT or MRI; (b) screened for cognitive dysfunction after stroke by the Montreal Cognitive Assessment (MoCA) (total scale score < 26); and (c) right-handed and aged between 18 and 80 years, regardless of sex, native Chinese, and years of education ≥ 6 years. The exclusion criteria were (a) unstable vital signs (blood pressure, heart rate, etc.,) or serious diagnosed primary diseases that are not suitable for cognitive rehabilitation; (b) coma, severe cognitive dysfunction, severe motor dysfunction, complete aphasia or sensory aphasia and other patients who cannot actively cooperate with rehabilitation training; (c) skin lesions, inflammation or metal parts implants in the stimulation area; (d) self-rating depression scale (SDS) score ≥ 50 [26] or self-rating anxiety scale (SAS) score ≥ 50 [27]; and (e) participation in other clinical trials that may affect the final assessment results.

### Study design and settings

There were four treatment groups, namely, the conventional cognitive training (CCT) group, tDCS group, CACT group, and CACT combined with tDCS group. According to the random number table generated by SPSS statistical software and numbered, eligible subjects were randomly divided into four groups by a statistician with no knowledge of the study. To blind the outcome evaluators, the distribution results were hidden in opaque sealed envelopes.

### Conventional cognitive training group

The CCT was carried out using props (including, paper and pen, cards, building blocks, etc.) by the same experienced professional therapist. The training included (1) Orientation training: The patient was guided to answer the questions related to the task, time, place and orientations. When the difficulty needed to be increased, the patients could be asked to describe the daily life activities conducted in the previous day, including when, where and with whom to do what. (2) Attention training: The therapist said a string of random numbers or letters, commanded the patient to clap their hands when they hear a specific number or letter, and increased the difficulty by increasing the number or letter that needs attention. (3) Memory training: The therapist read out a string of numbers or letters and ask patients to repeat; To increase the difficulty, the therapist asked the patient to remember a sentence before the cognitive training, such as “I drank two glasses of milk last night”, and asked the patient to recall this sentence at the end of training. (4) Calculation training: Cards with numbers were used for patients to calculate or compare sizes. (5) Executive function training: The patient was asked to copy the graph, which can be changed from a plane plan to a stereogram or a physical object by changing the complexity of the pattern. Patients were asked to classify the different categories of cards that were mixed together (people, animals, buildings, natural scenery, etc.). A total of 15 training sessions were held over three weeks, once daily for 5 days per week. Usually, each session was performed within 20 min, but the content and difficulty of the training could be adjusted by a professional therapist based on the patient’s specific cognitive status.

### Transcranial direct current stimulation group

The left DLPFC, which is closely related to PSCI [16, 28], was used as the stimulation area, and a 2 cm×3 cm anode electrode sheet was placed on the region located by 10–20 EEG. The cathode electrode was placed on the right supraorbital region [17]. The current intensity was 2.0 mA, and each treatment lasted 20 min, 5 times a week, for 3 weeks. The treatment was performed by the same rehabilitation therapist using the same machine (VOLGAN VC-8000 F, Nanjing Volgan Medical Technology Co., Ltd). The treatment was supervised by a professional neuroregulatory therapist.

### Computer-aided cognitive training group

The computer-aided cognitive rehabilitation training system (*66nao Brain rehabilitation system, China*) was carried out on a tablet computer with built-in intelligent electronic brain fitness cloud service software. A total of 161 cognitive training game scenarios in different fields and difficulty levels were built into the software. For instance, “judging the direction” and “looking for treasure were for orientation training”; “Moving point click” and whack-a-mole for attention training; “Fruit and vegetable paradise” (Patients remember fruits and vegetables in random order) for memory; “Poker sum”, “poker comparison” for calculation; And “express packing” and “tool chest” for executive function. A total of 15 training sessions were held over three weeks, once daily for 5 days per week. Each session was 20 min. Each gamelasted approximately one minute, so there were 20 random games were played in a single training session. The software customized personalized algorithms based on basic palliative information such as age, sex, education level, disease diagnosis, and cognitive assessment results. This makes it possible to intelligently customize training plans for patients, rather than simply training various brain games. The difficulty of the training process was dynamically adjusted in real time, and when the accuracy and speed of the patient reached a certain level, the difficulty of the game was automatically increased, which always matched the current cognitive level of the patient. Score feedback was provided for each training session to help the therapist understand patients’ level of cognition and improvement. Before the first training session, a dedicated therapist was responsible for teaching the patient how to use CACT, and the therapist supervised but didn’t participate in the treatment during the training.

### Computer-aided cognitive training group combined with tDCS group

During CACT training, tDCS stimulation of the left DLPFC was simultaneously performed for 20 min each session. The treatment frequency was once daily for 5 days per week for 3 weeks. The scheme of CACT and tDCS was the same as above.

### Measurements

Patients were assessed and examined before and after a 3-week intervention by the same experienced therapist and sonographer who were blind to the participants’ groups.

#### Cognitive function

MoCA shows the acceptable responsiveness and criterion validity in patients with PSCI [29]. An increase in MoCA scores above the minimal clinically importance difference of 1.22 predicts a significant improvement in cognition [29]. MoCA [30] included 8 perspectives: visuospatial and executive ability, naming, memory, attentional computation, language, abstraction, delayed memory, and orientation. The scale ranges from 0 to 30. A total score of less than 26 indicated the presence of cognitive function impairment, and a lower score suggested more severe cognitive impairment.

### Activities of daily living

The Instrumental Activities of Daily Living Scale (IADL) [31] was used to evaluate quality of life, and there were nine subitems of complex daily activities: telephone use, going out, shopping, food cooking, household maintenance, furniture repair, laundry, taking medicine, and financial management. The scale ranged from 0 to 27 points. A higher score indicated a better ability to perform activities of daily living.

### Cerebrovascular function

The transcranial Doppler ultrasound (TCD) (TCD-2000 S, Beijing Chioy Medicial Technology Co., Ltd), a noninvasive diagnostic tool that can reflect changes in brain perfusion, was used to evaluate cerebrovascular function. A posterior temporal window was selected to detect the middle cerebral artery (MCA) using a 2 MHz TCD probe at a depth between 25 and 50 mm [32]. The systolic flow velocity (V_s_) and diastolic flow velocity (V_d_) of the MCA were measured, and the velocity of mean cerebral blood flow (V_m_) was calculated automatically by a computer using the following formula: V_m_=(Vs+(V_d_×2)/3 [33].

Cerebral blood flow velocity (CBFV) was expressed as the maximum blood flow velocity of the MCA. Pulse index (PI) [33] and breath holding index (BHI) [34] reflected the resistance of the blood vessels and cerebral vasomotor function. PI was displayed directly by the instrument and automatically calculated with the following formula: PI = (Vs − Vd)/Vm. Measuring BHI required patients to hold their breath for 30 s; then, a 2 MHz TCD probe was used through the temporal window [34], the Vm of the MCA is recorded before (V_m1_) and after breath holding (V_m2_), and the time for breath holding and calculating the BHI volume are performed with the following formula [35].

$$\mathbf{B}\mathbf{H}\mathbf{I}=\frac{({\varvec{V}}_{\varvec{m}2} - {\varvec{V}}_{\varvec{m}1})\varvec{*}100}{{\varvec{V}}_{\varvec{m}1}\mathbf{*}\varvec{T}\varvec{i}\varvec{m}\varvec{e}}$$

### Statistical analysis

A statistician unaware of the allocation groups was responsible for collecting and analysing patient data. The Shapiro–Wilk test was used for assessing the normality of all the data. The chi-square test, ANOVA or rank sum test was used to compare baseline data between groups according to the type and distribution of the data. When a normal distribution was detected, the paired *t* test was used for intragroup comparisons before and after treatment, ANOVA was used to analyse the difference in efficacy between groups after treatment, and the LSD post hoc test was used for pairwise comparisons. Alternatively, the Wilcoxon signed-rank test and Kruskal‒Wallis *H* test were used for data with nonnormal distributions or uneven variance, and Bonferroni pairwise comparisons were conducted. *P* < 0.05 was considered to indicate statistical significance. All the statistical analyses were conducted using SPSS 26.0 software (version 26.0, Chicago, IL, USA).

### Sample size calculation

According to previous literature reports [36], the mean MoCA score in the monotherapy group was 15.4, the standard deviation was 1.25, and that in the combined treatment group was 1.5 points greater than that in the monotherapy group, with similar variances. We obtained a single group of 15 patients for which the specific formula was used [37] (α = 0.05, β = 0.20). A total of 72 participants were included in the four groups, for a total of 20%.

## Results

### Patient demographic characteristics

**Fig. 1:**
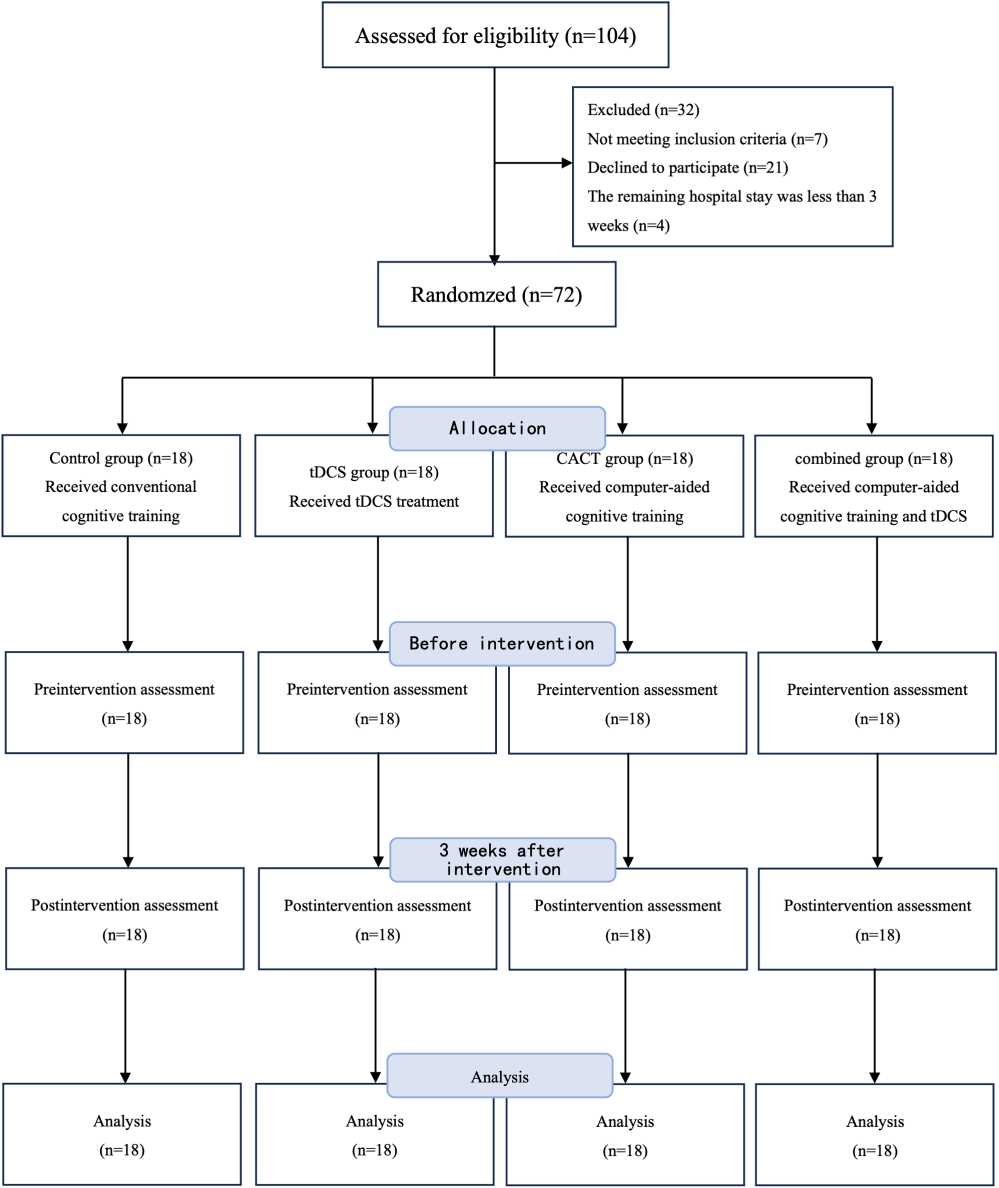
Flow chart of the study

There were 104 patients with PSCI considered for recruitment from the department of rehabilitation medicine of West China Hospital from December 2021 to September 2022. Seven of whom did not meet the criteria, 21 refused to participate and 4 were excluded because the expected hospital stay was not enough to complete the full course of treatment. Finally, 72 participants provided informed consent for the study and completed the full experiment (Fig. 1). There was no significant difference in the baseline data within four groups (*n* = 18 in each group; *P* > 0.05; Table 1).

**Table 1:** Comparison of baseline data among the four groups of subjects

Characteristic	CCT group	tDCS group	CACT group	CACT + tDCS group	*X2/F/Z*	*P*
Gender, n						
Male	11	13	12	13	0.238^a^	1.000
Female	7	5	6	5		
Age, yr	65.17 ± 3.26	58.50 ± 3.75	62.44 ± 2.76	61.06 ± 3.08	0.74^b^	0.532
BMI, kg/m2	23.88 ± 3.34	25.56 ± 2.93	23.36 ± 3.36	24.53 ± 3.26	1.333^b^	0.273
Affected hemisphere, n						
left	5	7	9	11	0.213^c^	1.000
right	13	11	9	7		
Duration of stroke, day, median [IQR]	29.5(15.3–95.8)	31.0(24.5–74.5)	38.5(12.5–99.3)	22.5(16.5–22.5)	1.569^c^	0.666
Education level, yr, median [IQR]	9(6–16)	12(8.3–15)	9(9–15)	12(9–15)	0.511^c^	0.916
SAS	28.89 ± 1.16	29.94 ± 1.39	29.33 ± 1.43	27.39 ± 1.19	0.732^b^	0.563
SDS	29.28 ± 1.42	31.89 ± 1.24	31.39 ± 1.29	31.17 ± 1.04	0.824^b^	0.485

IQR: interquartile range; *P* value: a comparison between four groups

^a^ Analyzed by the χ2 test; ^b^Analyzed by one-way ANOVA test; ^c^Analyzed by the Kruskal‒Wallis test

CCT: conventional cognitive training; tDCS: transcranial direct current stimulation; CACT: computer-aided cognitive training; BMI: Body mass index

### Cognitive function

The MoCA scores before treatments did not significantly differ among the group (*P* = 0.64), but all the groups increased the MoCA scores after treatment (*P* = 0.008). There were differences in MoCA score improvement among four groups (*F* = 11.346, *P* < 0.0001). The LSD post hoc test showed that there was no significant difference in efficacy among CCT, tDCS and CACT groups. CACT combined with tDCS could increase MoCA scores much more than the other groups (*P* < 0.0001; *P* = 0.0001; *P* < 0.0001), suggesting that CACT combined with tDCS group was significantly better at improving cognitive function (Table 2).

**Table 2:** Intragroup and intergroup comparisons of MoCA scores in the four groups

Groups		MOCA			within group Difference^1^		P (Between- Groups Difference^2^)	
		Pre	Post	Diff	t	P	Comparing to group 1	Comparing to group 4
CCT group		14.50 ± 5.70	16.89 ± 6.22	2.39 ± 1.75	-5.78	< 0.0001*	-	< 0.0001*
tDCS group		12.72 ± 6.32	16.06 ± 7.57	3.33 ± 4.35	-3.249	0.005*	0.391	0.0001*
CACT group		14.89 ± 5.83	17.28 ± 5.84	2.39 ± 2.91	-3.479	0.003*	1.000	< 0.0001*
CACT + tDCS group		14.78 ± 4.93	22.61 ± 4.39	7.83 ± 3.54	-9.4	< 0.0001*	< 0.0001*	-
Between- Groups Difference^3^	*F*	0.565	4.276	11.346				
	*P* value	0.64	0.008*	< 0.0001*				

Diff: difference; group1: CCT group; group 4: CACT + tDCS group; CCT: conventional cognitive training; tDCS: transcranial direct current stimulation; CACT: computer-aided cognitive training;

1: paired t test; 2: LSD post hoc test; 3: one-way ANOVA test;

* Statistically significant (*P* < 0.05)

The subdomains of MoCA were analysed, which revealed that attentional computation and delayed memory were improved in the CCT group (*P* = 0.007; *P* = 0.005). The tDCS group improved three subprojects, naming, attentional computation and delayed memory (*P* = 0.023; *P* = 0.013; *P* = 0.011). The CACT group improved delayed memory for only one subproject(*P* = 0.007). And all subdomains including visuospatial and executive ability, naming, attentional computation, language, abstraction, delayed memory and orientation were improved in the CACT combined with tDCS group (All *P* < 0.05; Table 3). The difference in sub-scores before and after treatment among the four groups indicated that the CACT combined with tDCS group could improve visuospatial and executive ability more than the other three groups (*P* < 0.0001; *P* = 0.0001; *P* = 0.0028). In attentional computation, the CACT combined with tDCS group improved more than the CACT group did (*P* = 0.0011). In terms of language improvement, the CACT combined with tDCS group was superior to the tDCS group (*P* = 0.0011). There were no differences among the four groups in the other subdomains (*P* > 0.05).

**Table 3:** Subdomain analysis of the MoCA scores in the four groups

Group		MoCA subdomains						
		visuospatial and executive ability	naming	attentional computation	language	abstraction	delayed memory	orientation
CCT group	Pre	1(0,2.25)	3(1,3)	4(2,5.25)	1(0,2.25)	1(0,1)	0(0,1)	4(3,6)
	Post	1(0,3)	3(2,3)	5(3,6)	1.5(0,3)	1(0,2)	1(0,2)	5(4,6)
	Diff	0(0,0)^a^	0(0,0.25)	0(0,1)	0(0,0.25)	0(0,1)	0.5(0,1)	0(0,1)
	*Z*	-1.000	-1.857	-2.714	− 0.816	-1.633	-2.810	-1.897
	*P*	0.317	0.063	0.007*	0.414	0.102	0.005*	0.058
tDCS group	Pre	1(0.75,3)	2(0,3)	2(0.75,5)	1(0,2)	1(0,1.25)	0(0,1)	4(2,5)
	Post	1(1,2.25)	2(0.75,3)	5(2,5)	0.5(0,3)	1(0,2)	1(0,3)	5(2.75,6)
	Diff	0(0,1)^a^	0(0,1)	1(0,2)	0(-0.25,0)^a^	0(0,0)	0.5(0,2)	0(0,2.25)
	*Z*	− 0.933	-2.271	-2.480	− 0.431	-1.633	-2.547	-1.379
	*P*	0.351	0.023*	0.013*	0.666	0.102	0.011*	0.168
CACT group	Pre	1(0,2.25)	2.5(1,3)	4.5(2.75,5)	1(0,2)	1(0,2)	0(0,1)	4.5(3.5,6)
	Post	1.5(1,3.25)	3(1,3)	4(3.75,5)	1(0.75,2)	1(0.75,2)	1(0,3.25)	4.5(4,5.25)
	Diff	0(0,1)^a^	0(0,1)	0(0,1)^a^	0(0,1)	0(-0.25,0.25)	0.5(0,2)	0(-0.25,1)
	*Z*	-1.492	-1.265	-1.512	-1.310	− 0.302	-2.699	− 0.489
	*P*	0.136	0.206	0.131	0.19	0.763	0.007*	0.625
CACT + tDCS group	Pre	1(0,2)	2(1.75,3)	3.5(2,5)	1(0,2)	1(1,1)	0(0,1.25)	4.5(3,6)
	Post	3(2,4)	3(2.75,3)	5.5(5,6)	2(1.75,3)	1(1,2)	3(1,3)	6(4.75,6)
	Diff	1.5(1,2.25)	1(0,1.25)	2(0.75,2.25)	1(0,1)	0(0,1)	1.5(0,3)	1(0,2)
	*Z*	-3.453	-2.873	-3.347	-2.913	-2.333	-2.971	-2.672
	*P*	0.001*	0.004*	0.001*	0.004*	0.02*	0.003*	0.008*

CCT: conventional cognitive training; tDCS: transcranial direct current stimulation; CACT: computer-aided cognitive training; *Z*: Wilcoxon signed-rank test; * Statistically significant (*P* < 0.05); ^a^ compared to the CACT combined with tDCS group, *P* < 0.05

### Instrumental activities of daily life

There was no difference in IADL scores before inventions of the four groups. The IADL differences before and after treatments were significantly different among the four groups (*F* = 32.262, *P* < 0.0001), and pairwise comparisons suggested that the combined treatment group had better IADL improvements than did the other three groups (*P* < 0.0001, *P* < 0.0001, *P* = 0.022), indicating that the combined treatment group was significantly better at improving activities of daily living (Table 4).

**Table 4:** Intragroup and intergroup comparisons of IADL scores in the four groups

Groups		IADL			within group Difference^1^		*P* (Between- Groups Difference^2^)	
		Pre	Post	Diff	*T*	*P*	Comparing to group 1	Comparing to group 4
CCT group		6.00(2.50,6.00)	6.00(3.75,8.00)	0.00(0.00,2.00)	-2.399	0.016*	-	< 0.0001*
tDCS group		5.50(1.75,11.00)	8.50(6.00,13.25)	3.00 (0.00,4.25)	-3.192	0.001*	0.113	< 0.0001*
CACT group		6.00(2.75,9.75)	7.00(3.00,12.00)	1.00 (0.00,2.00)	-2.654	0.008*	1.000	0.022*
CACT + tDCS group		5.50(3.00,11.50)	17.00(9.00,22.50)	5.50(3.75,14.00)	-3.73	< 0.0001*	< 0.0001*	-
Between groups^3^	*H*	1.66	5.986	32.262				
	*P*	0.203	0.002*	< 0.0001*				

CCT: conventional cognitive training; tDCS: transcranial direct current stimulation; CACT: computer-aided cognitive training; Diff: difference; group1: CCT group; group 4: CACT combined with tDCS group;

^1^ Wilcoxon signed-rank test; ^2^ Bonferroni post hoc test; ^3^ Kruskal‒Wallis H test

* Statistically significant (*P* < 0.05)

Subdomain analysis of IADL was also conducted. The results showed that there was no significant difference between the CCT group and the CCT group in each subitem after treatment (All *P* > 0.05). The scores for food cooking, household maintenance, laundry and financial management in the tDCS group all increased after treatment (*P* = 0.008; *P* = 0.021; *P* = 0.021; *P* = 0.007). The financial management subitems in the CACT group improved after treatment (*P* = 0.014), and all subitems in the CACT combined with tDCS group were significantly increased after treatment (All *P* < 0.05; Table 5). The difference before and after treatment among the four groups showed that the combined treatment group was better at improving the shopping scores than the other three groups (*P* < 0.001; *P* = 0.002; *P* = 0.011). In terms of household maintenance, furniture repair and laundry, the scores of the combined treatment group increased much more than those of the CCT group and CACT group (All *P* < 0.01). In improving food cooking and financial management, the CACT combined with tDCS group performed better than the CCT group did (*P* = 0.023; *P* = 0.002). In medication use, the CACT combined with tDCS group was better improved than the tDCS group (*P =* 0.017). There was no difference in the improvement in telephone use among the four groups (*P* > 0.05).

**Table 5:** Subdomain analysis of IADL scores in the four groups

Groups		IADL subdomains								
		telephone use	going out	shopping	food cooking	household maintenance	furniture repair	laundry	taking medicine	financial management
CCT group	Pre	3(0.75,3)	0(0,0)	0(0,0)	0(0,0)	0(0,0)	0(0,0)	0(0,0)	1.5(0,3)	0(0,1.25)
	Post	3(1.75,3)	0(0,0)	0(0,0)	0(0,0)	0(0,0.25)	0(0,0)	0(0,0.25)	2.5(0.75,3)	0(0,2.25)
	Diff	0(0,0)	0(0,0)^a^	0(0,0)^a^	0(0,0)^a^	0(0,0)^a^	0(0,0)^a^	0(0,0)^a^	0(0,0.25)	0(0,0)^a^
	*Z*	-1.342	0.000	-1.414	-1.000	-1.414	0.000	0.000	-1.414	-1.414
	*P*	0.18	1	0.157	0.317	0.157	1	1	0.157	0.157
tDCS group	Pre	3(0,3)	0(0,1)	0(0,0)	0(0,0)	0(0,0.25)	0(0,0)	0(0,0)	2(1,3)	0(0,1)
	Post	3(2.25,3)	1(0,1)	0(0,1)	1(0,1)	1(0,1)	0(0,0.25)	1(0,1)	2.5(0,3)	1(0,2)
	Diff	0(0,0)	0(0,0.25)^a^	0(0,0)^a^	0(0,1)	0(0,1)	0(0,0)	0(0,1)	0(0,0)^a^	0(0,1)
	*Z*	-1.633	− 0.707	-1.633	-2.646	-2.309	-1.732	-2.308	− 0.276	-2.714
	*P*	0.102	0.48	0.102	0.008*	0.021*	0.083	0.021*	0.783	0.007*
CACT group	Pre	3(1.75,3)	0(0,0.25)	0(0,1)	0(0,0)	0(0,0)	0(0,0)	0(0,0)	2(1,3)	0(0,1.25)
	Post	3(2,3)	0(0,1)	0(0,1)	0(0,1)	0(0,1)	0(0,0)	0(0,0)	2.5(1,3)	1(0,2.25)
	Diff	0(0,0.25)	0(0,0)^a^	0(0,0)^a^	0(0,0)	0(0,0)^a^	0(0,0)^a^	0(0,0)^a^	0(0,1)	0(0,1)
	*Z*	-1.414	-1.414	-1.000	-1.414	-1.732	0.000	− 0.816	-1.508	-2.460
	*P*	0.157	0.157	0.317	0.157	0.083	1	0.414	0.132	0.014*
CACT+ tDCS group	Pre	3(2,3)	0(0,1)	0(0,1.25)	0(0,0.5)	0(0,0.25)	0(0,0)	0(0,0.25)	2(1,3)	0(0,1.25)
	Post	3(3,3)	1(0,3)	1(0,3)	1(0,3)	2(1,2)	0(0,1)	1(0,3)	3(2.75,3)	2(1,3)
	Diff	0(0,1)	1(0,1.25)	1(0,1)	0(0,1.25)	1(0.75,2)	0(0,1)	1(0,2)	1(0,1.25)	1(0,2.25)
	*Z*	-2.264	-2.801	-3.066	-2.558	-3.376	-2.530	-2.850	-3.022	-3.275
	*P*	0.024*	0.005*	0.002*	0.011*	0.001*	0.011*	0.004*	0.003*	0.001*

CCT: conventional cognitive training; tDCS: transcranial direct current stimulation; CACT: computer-aided cognitive training; *Z*: Wilcoxon signed-rank test; * Statistically significant (*P* < 0.05); ^a^ compared to the CACT combined with tDCS group, *P* < 0.05

### Cerebrovascular function

Intragroup comparisons of CBFV and PI before and after treatment among the four groups revealed no significant differences (*P* > 0.05). Only the CACT combined with tDCS treatment group significantly increased BHI after treatment (*Z*=-2.509, *P* = 0.012), and the other three groups did not improve BHI (*P* > 0.05; Table 6).

**Table 6:** Intragroup comparisons of MFV, PI and BHI among the four groups

Groups		CBFV	PI	BHI
CT group	Pre	52.28(40.28,64.93)	1.00(0.89,1.10)	0.70(0.45–0.89)
	Post	50.75(42.96,65.84)	1.08(0.76,1.18)	0.81(0.62–1.10)
	*Z*	-0.155	-1.035	-0.663
	*P*	0.877	0.301	0.508
tDCS group	Pre	58.30(44.80,69.05)	0.94(0.75,1.02)	0.54(0.37–0.73)
	Post	51.15(44.20,71.40)	0.87(0.70,1.05)	0.66(0.52–0.86)
	*Z*	-0.227	-1.534	-1.962
	*P*	0.82	0.125	0.050
CA group	Pre	58.43(49.23,82.43)	0.89(0.71,1.13)	0.48(0.37–0.67)
	Post	56.28(48.79,69.23)	0.90(0.74,1.19)	0.70(0.47-1.00)
	*Z*	-1.761	-0.621	-1.136
	*P*	0.078	0.535	0.256
CACT + tDCS group	Pre	51.55(40.33,61.88)	1.02(0.85,1.12)	0.59(0.46–0.82)
	Post	53.80(,39.80,65.30)	0.98(0.79,1.13)	0.83(0.64–1.02)
	*Z*	-0.207	-0.213	-2.509
	*P*	0.836	0.831	0.012*

CBFV: cerebral blood flow velocity;

PI: pulse index; BHI: breath holding index; Z: Wilcoxon signed-rank test;

* Statistically significant (*P* < 0.05)

### Adverse effects

During the trials, only one subject experienced skin redness after the first tDCS treatment. However, the subject expressed a willingness to continue the treatment after being treated by a professional doctor. No similar situation occurred after the current intensity was reduced in the subsequent treatment.

## Discussion

Although compared to CCT, both CACT and tDCS didn’t show greater improvement in cognitive impairment and the ability of daily living in stroke patients, we still recommended CACT and t DCS as the substitute of CCT, because CACT was the most attractive intervention in recent years with interesting games [38] and tDCS was low cost, non-invasive and easy to operate [13]. Simultaneous synergies between CACT and tDCS may arise as our study found that CACT combined with tDCS showed greater advantage on whole function and subdomains of cognitive function and activities of daily living ability. CACT can activate multiple brain regions associated with cognition, such as DLPFC and the posterior cingulate cortex, and enhance recruitment of brain networks [39]. Lisanne’s study found that improved cognitive function is associated with reduced correlation between the default mode network and the fronto-parietal network, and CACT can effectively reduce this correlation [40]. Rosaria believed that CACT could promote neuroplasticity through interesting, repetitive, multisensory stimulating tasks [41]. A study showed that CACT combined with tDCS can improve PSCI, proving that “CACT plus” was a promising tool for the treatment of PSCI [42]. Adding tDCS in CACT can cause bilateral prefrontal excitatory changes [43], improve cerebral microcirculation, increase blood oxygen levels, improve oxygen supply and diffusion in the damaged area, accelerate the functional recovery in the damaged brain area [44–46], and thus improve cognitive function. Since IADL function is affected by cognitive function, patients’ daily life is also well improved [47].

The changes of blood supply to the brain and cerebral hemodynamics may be related to the improvement of cognitive status [48, 49]. Although our study did not show an improvement in CBFV and PI, we found that CACT combined with tDCS group showed improvement in vasomotor function after treatment, reflected by an increase in BHI. That is consistent with the findings of previous studies [50–52]. A study revealed that when cognitive tasks were performed, activation of the DLPFC was significantly increased, as detected by functional near-infrared spectroscopy [53], which suggested that cognitive function was related to the supply of blood and oxygen to the frontal lobe. Another study also suggested that the MoCA score was positively correlated with regional cerebral blood flow in the prefrontal-subcortical circuits [24]. Neuromodulation may regulate neurovascular coupling in the ischemic population [54], Ryan et al. found that the improvement of cognition with tDCS was accompanied by changes in the hemodynamic response of the DPC and ventral medial prefrontal cortex, suggesting a mechanism by which tDCS can alter neuronal activity tendencies during cognitive tasks [55]. Therefore, we believe that changes in BHI in the CACT combined with tDCS group may enable blood vessels to distribute more blood flow to cognitively related brain regions such as the frontal lobe.

Studies have shown that no less than 77% of visuoexecutive deficits can be observed in stroke patients [4]. Our results suggested CACT combined with TDCS a promising intervention for visuoexecutive deficits after stroke. The mechanism of visuospatial and executive dysfunction is unknown and may be associated with the prefrontal cortex and its subcortical circuits [56]. Katrine et al. believed that CACT could effectively improve the visual space disorder after stroke [57]. Wang Z et al. [58]. applied 2 mA anode tDCS to the DLPFC, using neuropsychological scales to monitor the executive function of patients after stroke, and found that the executive function of stroke patients improved. These studies support CACT combined with tDCS as an effective treatment for visuospatial and executive dysfunction after stroke.

Learning to use computers for cognitive rehabilitation did not seem to be a difficult task for people with brain injuries [59]. Moreover, there has been a study supporting the efficacy and safety of home transcranial direct current [15]. In addition, serious side effects did not appear in our study, so combining CACT and tDCS may be of interest to patients with mobility difficulties, those who wish to recover at home or other populations. The present study seems to be the first assessment of cerebrovascular function using TCD in individuals subjected to PSCI, and we drew the conclusions that CACT or tDCS can’t influence cerebral blood supply velocity or vascular resistance outcomes, but vasomotor function may change when CACT and tDCS are combined, which affirmed the application value of TCD in related fields. For the reason that cerebrovascular reactivity is suggested to be the earliest detectable hemodynamic parameter related to cognition [60], we believe that BHI may have some value in cognitive assessment.

Through our study, we recommend the combined use of CACT combined with tDCS in the overall cognitive and activities of daily living ability. The specific effects of CACT combined with tDCS on visuospatial and executive ability need to be further studied because there was insufficient evidence before. The role of the identified vasomotor changes in cognitive improvement after stroke was unclear and requires further study. In addition, this study also leaved much to be desired: There was a lack of more objective tests for the assessment of cognitive function and follow-up data concerning the long-term effects of these therapies. Therefore, a subsequent trial should be planned.

## Conclusion

Both CACT and tDCS improved cognitive function and activities of daily living ability after stroke and could be used as an alternative to CCT therapy. CACT combined with tDCS showed additional benefits, which might be associated with improvement of cerebral vasomotor function. Besides, CACT combined with tDCS might be a promising method for visual spatial execution disorder.

## Data Availability

The datasets supporting the conclusion of this article are included within the article.

## Abbreviations

BHI: Breath-Holding Index
CACT: Computer-aided cognitive training
CBFV: Cerebral blood flow velocity
CCT: conventional cognitive training
DLPFC: left dorsolateral prefrontal cortex
IADL: Instrumental activities of daily living scale
MCA: middle cerebral artery
MoCA: Montreal cognitive assessment
PI: pulse index
PSCI: poststroke cognitive impairment
SAS: self-rating anxiety scale
SDS: self-rating depression scale
tDCS: transcranial direct current stimulation
TCD: transcranial Doppler ultrasound
